# Case report: Spontaneous coronary artery rupture presenting with acute coronary syndrome: A rare diagnosis of common disease

**DOI:** 10.3389/fcvm.2022.922180

**Published:** 2022-08-11

**Authors:** Ahmed Ibrahim Sayed

**Affiliations:** Faculty of Medicine, Jazan University, Jizan, Saudi Arabia

**Keywords:** coronary disease, acute coronary syndrome, spontaneous coronary rupture, surgery, pericardial effusion

## Abstract

Acute coronary syndrome (ACS), myocardial infarction, and sudden death have all been linked to spontaneous coronary artery rapture (SCAR). Patients primarily afflicted by SCAR are those with or without cardiovascular risk factors, notably men, implying a mechanism distinct from the more prevalent atherosclerosis. Both medical and interventional treatment should consider the diverse causes of ACS as well as the patient's clinical stability. I herein report an unusual case of a 33 years old male who presented with acute chest pain to the emergency department. His physical exam was normal. The electrocardiogram showed non-specific ST segment changes in anterior leads, and the echocardiogram revealed mild anterior wall hypokinesia with no evidence of pericardial effusion. He underwent coronary angiography which revealed a contained rupture in the anterior descending coronary artery. The patient underwent uneventful lifesaving coronary artery perforation repair. It concluded that, though rare, SCAR should be considered as a differential diagnosis in patients with ACS, even in the absence of pericardial effusion in adult patients of all ages.

## Introduction

A coronary artery spontaneous rupture is a rare pathology that can manifest in different ways. In this sense, it can range from simple acute coronary syndrome (ACS) to cardiogenic shock secondary to large precordial effusion. Early detection and proper management can save the patient's life. Few case reports have been published that indicate its different presentations and variety of management approaches.

## Case presentation

**Figure 1:**
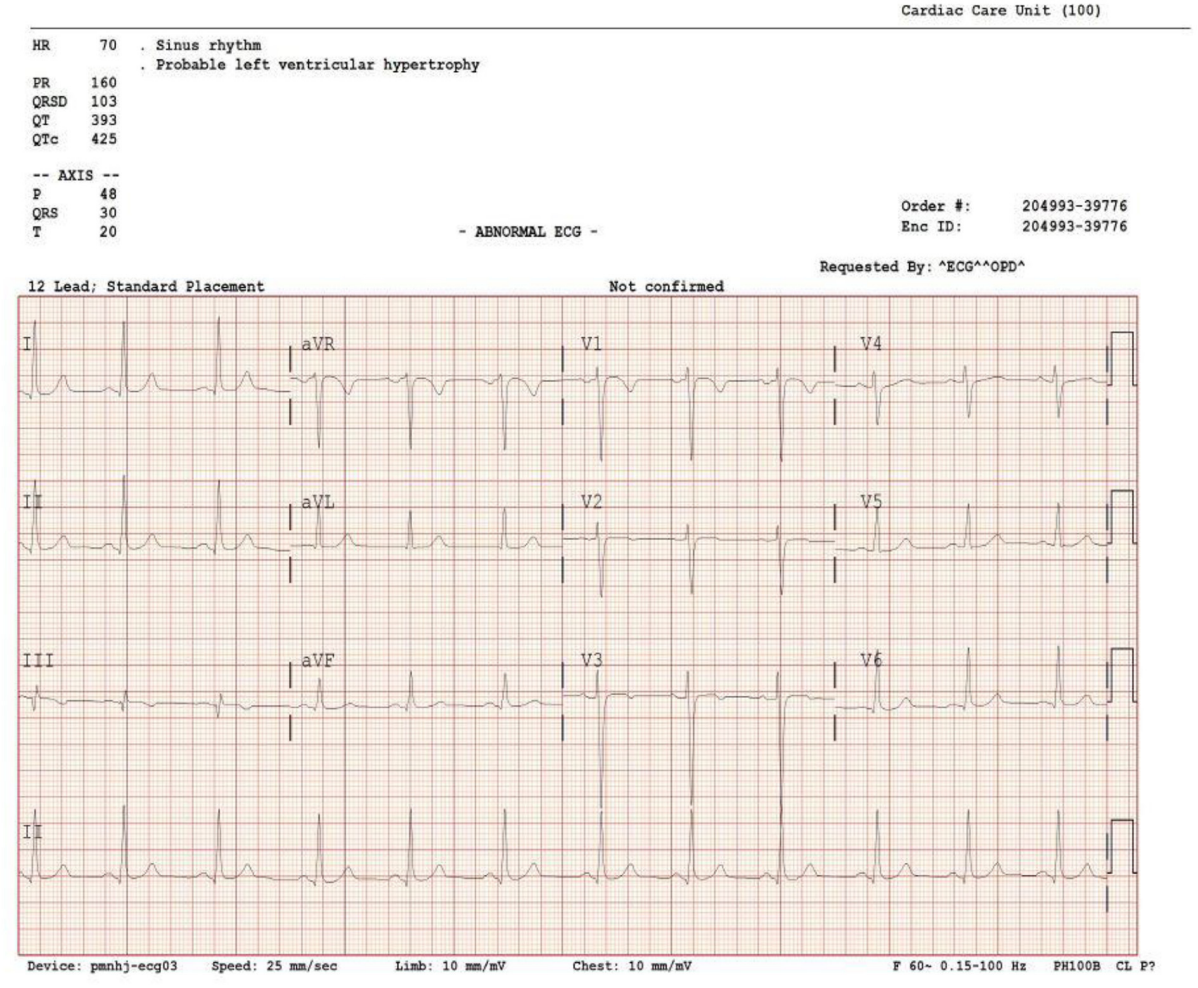
A 12-lead electrocardiogram shows non-specific ST-segment changes in anterior leads.

A 33-year-old male teacher with a history of smoking presented to the emergency department complaining of retrosternal chest pain for 2 days. It was a dull ache that radiated to his back. The pain was aggravated by minimal physical activity with no relieving factors. It was associated with mild fatigue. He had no fever, dyspnea, orthopnea, or syncope, and he did not exhibit any palpitation. He had no joints pain, rash or mouth ulcers, and no history of any chest trauma. The blood pressure (BP) was 125/65 mmHg, heart rate (HR) was 78 bpm, and saturation was 98% on room air. Cardiovascular examination was within normal standards, and no abnormal findings were recorded upon chest examination. His ECG demonstrated normal sinus rhythm with non-specific ST changes in anterior leads (Figure 1). Cardiac enzyme troponin I was 6.4 ng/mL (normal range <0.04 ng/mL). Low-density lipoprotein (LDL) measured 133 mg/dl (0–140 mg/dl), and total cholesterol was 215 mg/dL (0–239 mg/ dL). ESR 13 mm/h (normal 1–20 mm/h), C-reactive protein 2.9 mg/L (normal <10 mg/L). ANA, rheumatoid factors and ANCA were negative. Complete blood count (CBC) and renal function tests were within normal ranges. Additionally, the chest X-ray was normal.

**Figure 2:**
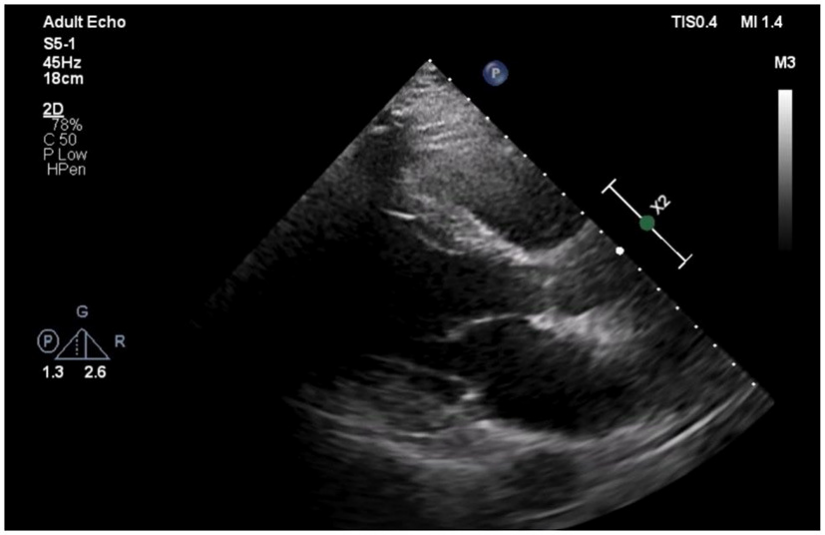
Echocardiogram of Left parasternal axis view shows no pericardial effusion.

**Figure 3:**
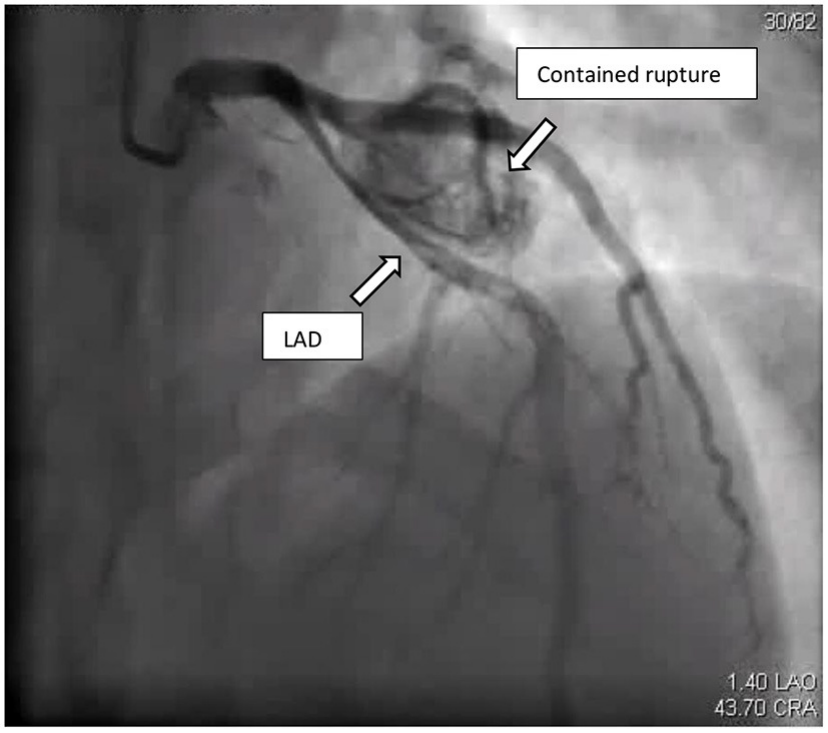
Coronary angiogram: cranial view, showed LAD with large contained rupture in proximal segment causing pressure effect on the LAD.

**Figure 4:**
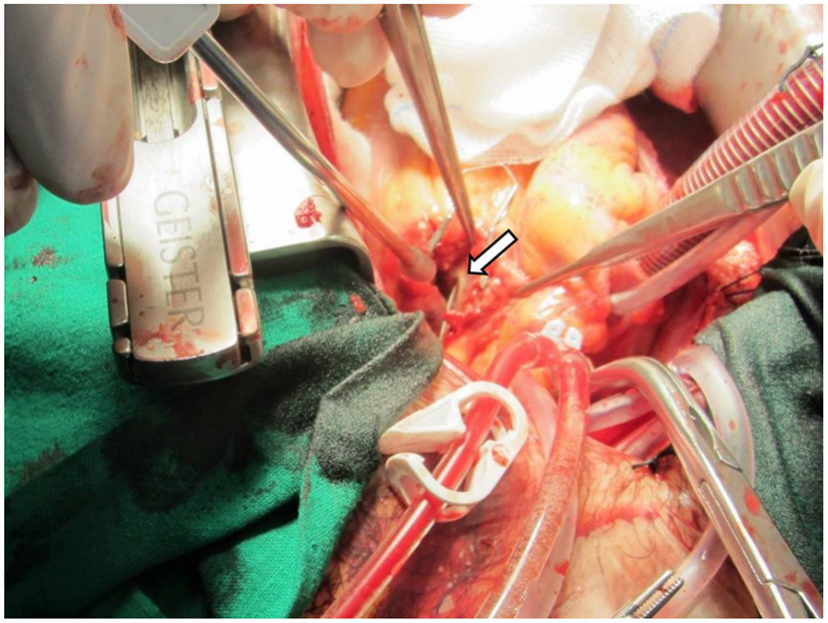
Intraoperative photo showing the LAD spontaneous rupture with probe inside the opening (arrow).

The patient was admitted to our coronary care unit as a case of ACS and was started on an ACS management protocol that includes aspirin, clopidogrel, statin, and subcutaneous heparin (enoxaparin 1 mg/kg twice daily). A transthoracic echocardiogram revealed a low normal ejection fraction (EF 50%) with mild anterior wall hypokinesia and no pericardial effusion; otherwise, no abnormality was detected (Figure 2). The patient was stabilized overnight and taken to the coronary catheterization laboratory for a coronary angiogram. The coronary angiogram showed normal left main (LM) artery, and the left anterior descending artery (LAD) exhibited a large pseudoaneurysm in proximal segment with contrast squirted in it and TIMI II-III flow in the distal LAD (Figure 3). The left circumflex artery (LCX) and the right coronary artery (RCA) were observed to be normal. The procedure was stopped, and the patient was urgently referred for a cardiac surgery consultation. Three hours later, he developed acute hypotension with a BP of 75/55 mmHg and HR of 133 bpm. Bolus IV fluid was given, and emergency bedside pericardiocentesis was performed in addition to the removal of 50 cc of fresh blood. The patient's BP stabilized, and he was taken as a lifesaving case to the operating room. Cardiopulmonary bypass (CPB) was established through the cannulation of the femoral vessels. A median sternotomy was performed, and 100 cc of fresh and clotted blood was removed from the pericardial sac. Then, careful examination of the pseudoaneurysm showed spontaneous rupture with contained bleeding. The opening of spontaneous rupture in the LAD was identified as shown in Figure 4, and it was closed with a 3–0 polypropylene continuous suture. Postoperatively, the patient did well. He did not show any signs of either myocardial infarction or left ventricle pump failure. Thus, he was discharged 6 days later in a stable condition.

## Discussion

Spontaneous coronary artery rupture (SCAR) is a rare condition that might present in different ways, some of which can mimic ACS. Given its potentially fatal consequences, it requires proper and emergency identification and management.

In a literature review, Longobardi et al. identified 8 case reports on SCAR (1). ACS was the most common presentation; however, some patients presented with hemodynamic instability due to large pericardial effusion. The majority of the cases reported involved middle-aged patients (50–70 years), except for 1 case that occurred in a patient younger than 40 years (37 years old) (1). Furthermore, the literature suggests that men are more likely to have this disease compared to women. In the reported cases, only one patient was female, and her age was 65 years (2). On another note, spontaneous coronary artery ruptures can happen at any major coronary artery and are rarely reported in the side branch (3, 4). Moreover, cardiogenic shock and tamponade complicate SCAR more often when it originates from RCA (5, 6).

Many conditions can be associated with arterial diseases that may lead to SCAR (such as atherosclerosis, Kawasaki disease, localized atheromatous plaque, aneurysm, blunt chest trauma, and very rarely, infection) (6–8). Of note, Kawasaki disease usually associated with giant calcified coronary aneurysms which might be complicated by perforation (9).

Coronary segment dilatation of more than 1.5% of the adjacent normal coronary segment is considered aneurysmal. Moreover, the prevalence of this pathology is from 0.3 to 5.3% according to early angiographic studies, the majority due to atherosclerosis (9). Common differential diagnoses of contained coronary perforation are true or pseudoaneurysm. A monolayer or double layer outwardly bulging within the coronary artery that lacks all 3 layers (intima, media, and adventitia) of the arterial wall is defined as pseudoaneurysm. This vessel wall integrity loss gives it more highly rupture-prone adventitia and perivascular tissue than true aneurysms. Pseudoaneurysm is commonly caused by traumatic dissection and perforation due to percutaneous coronary intervention (10). Differentiating true from pseudoaneurysm by plan coronary angiography is extremely difficult. However, using intravascular ultrasound (IVUS) imaging might help to delineate the aneurysm wall structure (11).

The site and size of SCAR in addition to the patient's condition can drive the management approach. Graft stenting through percutaneous coronary intervention is a reasonable option if the patient is stable and the anatomical location is appropriate (12). However, surgical intervention with pericardial evacuation must be performed when the patient is unstable or the anatomical location is not optimal (3).

My patient is unique because he was very young at 33 years of age. His only cardiac risk factor was smoking; he had no signs of inflammatory disease and no history of chronic disease or atherosclerosis, and no abnormality was detected by plain coronary angiogram a part from the perforation. Moreover, because there was no clear stenosis, atherosclerosis or aneurysm other than the perforation identified during the operation, the cardiac surgeon decided to repaired the perforation site only with no indications for bypass graft to LAD. Since I found no causative pathology, I suggested that this rupture was spontaneous.

## Conclusion

Spontaneous coronary artery perforation is a rare condition that could be underreported due to its lethal presentation. A high index of suspicion should be considered when a patient presents with acute coronary artery syndrome and pericardial effusion. Early diagnosis will lead to surgical or non-surgical intervention in a way that prevents its devastating complications.

## Learning points

- SCAR should be considered as a differential diagnosis in patients with acute chest pain, even in the absence of pericardial effusion.
- We should consider contained SCAR as one of the differential diagnoses when we observe large coronary pseudoaneurysm by coronary angiogram.
- In patients presenting with pericardial effusion, SCAR is a possible diagnosis.

## Data availability statement

The original contributions presented in the study are included in the article/supplementary material, further inquiries can be directed to the corresponding author.

## Author contributions

The author confirms being the sole contributor of this work and has approved it for publication.

## Conflict of interest

The author declares that the research was conducted in the absence of any commercial or financial relationships that could be construed as a potential conflict of interest.

## Publisher's note

All claims expressed in this article are solely those of the authors and do not necessarily represent those of their affiliated organizations, or those of the publisher, the editors and the reviewers. Any product that may be evaluated in this article, or claim that may be made by its manufacturer, is not guaranteed or endorsed by the publisher.

## References

[1] Longobardi A Iesu S Baldi C Di Maio M Panza A Mastrogiovanni G. Spontaneous coronary artery rupture presenting as an acute coronary syndrome evolved in pseudoaneurysm and cardiac tamponade: case report and literature review. Eur Heart J Acute Cardiovasc Care. (2017) 6:666–9. 10.1177/2048872615617043 26566773

[2] Hansch A Betge S Pfeil A Mayer TE Wolf G Brehm B. Images in cardiovascular medicine. Spontaneous rupture of the right coronary artery. Circulation. (2010) 121:2692–3. 10.1161/CIRCULATIONAHA.109.924290 20566967

[3] Kaljusto ML Koldsland S Vengen OA Woldbaek PR Tonnessen T. Cardiac tamponade caused by acute spontaneous coronary artery rupture. J Card Surg. (2006) 21:301–3. 10.1111/j.1540-8191.2006.00239.x 16684069

[4] Fujimoto D Takami M Kozuki A Shite J. A case report of unusual clinical features of a spontaneous coronary artery rupture: pathologic findings in the rupture site. Eur Heart J Case Rep. (2019) 3:1–6. 10.1093/ehjcr/ytz135 31384915 PMC6764561

[5] Shrestha BM Hamilton-Craig C Platts D Clarke A. Spontaneous coronary artery rupture in a young patient: a rare diagnosis for cardiac tamponade. Interact Cardiovasc Thorac Surg. (2009) 9:537–9. 10.1510/icvts.2009.207001 19491124

[6] Manzo-Silberman S Aelion H Leprince P. Spontaneous rupture of a coronary artery. Arch Cardiovasc Dis. (2014) 107:704–5. 10.1016/j.acvd.2012.09.007 23911832

[7] Dueholm S Fabrin J. Isolated coronary artery rupture following blunt chest trauma. A case report. Scand J Thorac Cardiovasc Surg. (1986) 20:183–4. 10.3109/14017438609106500 3738451

[8] Fan CC Andersen BR Sahgal S. Isolated myocardial abscess causing coronary artery rupture and fatal hemopericardium. Arch Pathol Lab Med. (1994) 118:1023–5. 7944886

[9] Yang EH Kapoor N Gheissari A Burstein S. Coronary and intracerebral arterial aneurysms in a young adult with acute coronary syndrome. Tex Heart Inst J. (2012) 39:380–3. 22719148 PMC3368465

[10] Kar S Webel RR. Diagnosis and treatment of spontaneous coronary artery pseudoaneurysm: rare anomaly with potentially significant clinical implications. Catheter Cardiovasc Interv. (2017) 90:589–97. 10.1002/ccd.26997 28258964

[11] Maehara A Mintz GS Ahmed JM Fuchs S Castagna MT Pichard AD. An intravascular ultrasound classification of angiographic coronary artery aneurysms. Am J Cardiol. (2001) 88:365–70. 10.1016/S0002-9149(01)01680-0 11545755

[12] Wiemer M Horstkotte D Schultheiss HP. [Non-surgical management of a perforated left anterior descending coronary artery following cardiopulmonary resuscitation]. Z Kardiol. (1999) 88:675–80. 10.1007/s003920050344 10525930

